# Age estimation during the blow fly intra-puparial period: a qualitative and quantitative approach using micro-computed tomography

**DOI:** 10.1007/s00414-017-1598-2

**Published:** 2017-05-04

**Authors:** Daniel Martín-Vega, Thomas J. Simonsen, Martina Wicklein, Martin J. R. Hall

**Affiliations:** 1 Department of Life Sciences, Natural History Museum, Cromwell Road, London, SW7 5BD UK; 2 Naturhistorisk Museum Aarhus, 8000 Aarhus C, Denmark; 3 Department of Neuroscience, Physiology and Pharmacology, University College London, London, WC1E 6BT UK

**Keywords:** Forensic entomology, Insect development, Metamorphosis, Morphology, Post-mortem interval, Pupal stage

## Abstract

Minimum post-mortem interval (_min_PMI) estimates often rely on the use of developmental data from blow flies (Diptera: Calliphoridae), which are generally the first colonisers of cadavers and, therefore, exemplar forensic indicators. Developmental data of the intra-puparial period are of particular importance, as it can account for more than half of the developmental duration of the blow fly life cycle. During this period, the insect undergoes metamorphosis inside the opaque, barrel-shaped puparium, formed by the hardening and darkening of the third instar larval cuticle, which shows virtually no external changes until adult emergence. Regrettably, estimates based on the intra-puparial period are severely limited due to the lack of reliable, non-destructive ageing methods and are frequently based solely on qualitative developmental markers. In this study, we use non-destructive micro-computed tomography (micro-CT) for (i) performing qualitative and quantitative analyses of the morphological changes taking place during the intra-puparial period of two forensically relevant blow fly species, *Calliphora vicina* and *Lucilia sericata*, and (ii) developing a novel and reliable method for estimating insect age in forensic practice. We show that micro-CT provides age-diagnostic qualitative characters for most 10% time intervals of the total intra-puparial period, which can be used over a range of temperatures and with a resolution comparable to more invasive and time-consuming traditional imaging techniques. Moreover, micro-CT can be used to yield a quantitative measure of the development of selected organ systems to be used in combination with qualitative markers. Our results confirm micro-CT as an emerging, powerful tool in medico-legal investigations.

## Introduction

Originally developed for its use in clinical practice [1], computed tomography (CT) was soon after applied to legal medicine [2]. Since then, the use of CT in post-mortem investigations has been progressively implemented in many institutions throughout the world [3]. Post-mortem CT in combination with conventional autopsy is currently recommended as the diagnostic gold standard for determining the cause of death [4]. Through these ‘virtual autopsies’ using post-mortem CT, even the entomological evidence associated with a cadaver can be analysed by locating and estimating the volume of maggot masses on the body [5].

Although still less available than CT to forensic practitioners, micro-computed tomography (micro-CT) can also be a powerful tool in medico-legal investigations [6]. Micro-CT is basically a microscope system based on the same principles of CT [7], thus providing much greater spatial resolution. In a forensic context, micro-CT has a wide range of potential roles, e.g. the examination of gunshot residue particles, the analysis of tool marks on bones or the analysis of the age of entomological evidence for the estimation of a minimum post-mortem interval (_min_PMI), among others [6, 8].

Estimations of a _min_PMI often rely on blow flies (Diptera: Calliphoridae) because they are generally among the first insects colonising cadavers, frequently within hours or even minutes after death [9]. The colonisation by blow flies starts when a female lays her eggs on the body; once the embryonic development is complete [10], the larvae will hatch and feed on the dead soft tissues, passing through three larval instars [11]. Fully grown third instar larvae stop feeding and usually disperse from the cadaver for pupariation. The third instar larva then contracts irreversibly, and its cuticle hardens and darkens, forming an opaque barrel-like case called the puparium (see [12] for more details on the mechanism of puparium formation). The insect will undergo metamorphosis inside the puparium, firstly through a pupal stage and then through the development of the pharate adult [13–16]. Once the histogenesis of the adult tissues is complete, the intra-puparial period ends with the emergence of the adult fly from the puparium [16]. As insects are poikilothermic organisms, the developmental rates of these different life stages are strongly influenced by the environmental temperature. Therefore, a _min_PMI can be accurately estimated on the basis of temperature information from the forensic scene and available development data for the pertinent species and the oldest developmental stage collected [9]. Developmental studies of the intra-puparial period are of particular importance in forensic entomology as it can occupy more than 50% of the duration required for the development of the blow fly [17, 18]. However, the opacity of the puparium makes the determination of age-specific measures or markers difficult, as there are virtually no observable external changes during the whole intra-puparial period. One method to address this is to open the puparium and extract the insect inside in order to identify the external morphological markers which are correlated with its age [8, 19, 20]. Although this is a relatively simple, cheap and valuable method, some of the potentially most informative characters are soft tissues which can be discoloured, displaced and/or disfigured during preservation or manipulation [20]. Moreover, some qualitative measurements of age, such as those based on colouration or degree of melanisation, are subjective by nature and are thus prone to errors in age estimations [20]. Furthermore, external morphological analyses do not enable an accurate delimitation of the different developmental stages (prepupa, pupa and pharate adult) occurring during the intra-puparial period, resulting sometimes in misinterpretations which may lead to significant errors in _min_PMI estimations [14]. Traditional histology might be, in this sense, much more informative of age-related internal morphological changes taking place during the intra-puparial development [19, 21]. However, it is also a significantly more time-consuming, destructive and invasive technique than just removing the puparial cuticle and, in the specific case of blow fly puparia, it usually results in fragmented sections due to the abundance of fat bodies and fatty droplets [16, 21]. This means a potential loss of significant parts of informative tissues. As a non-invasive technique, micro-CT can be an ideal alternative to traditional histology as simple staining methods provide high contrast imaging at similar spatial resolutions [22]. The potential use of micro-CT for ageing blow flies during the intra-puparial period was first explored by Richards et al. [8]. The results from that preliminary study show how tomographic virtual sections can provide enough resolution for qualitatively documenting the morphological changes in key organ systems at 25% intervals of the total intra-puparial period. Furthermore, the study strongly supported the possibility for achieving a more accurate temporal resolution for _min_PMI estimations with further research [8].

More than a century ago, Pérez [23] observed that “the temperature has a particularly active influence on the rapidity of the pupation phenomena, but there is a constant correlation between the [morphological] changes in the different organ systems”. This constancy of the chronology and relative timing of the morphological changes during metamorphosis at different temperatures allows for accurate ageing of puparial samples by correlating qualitative morphological markers to intervals or percentages of the total intra-puparial period [8, 20]. Nevertheless, it is always desirable to provide quantitative measures of age in combination with qualitative age-specific markers. Quantitative measures improve analysis objectivity and, consequently, the reliability of _min_PMI estimates [20]. Gene expression analysis [24] is certainly a promising and powerful quantitative ageing method, but it is also a destructive technique which precludes a subsequent morphological analysis of the sample. Micro-CT, on the other hand, has the potential to yield quantitative measures of organ growth, which may complement a qualitative morphological analysis [16]. This quantitative approach to the study of insect development using micro-CT was first described by Lowe et al. [25], who documented the changes in gut and tracheal volume during the metamorphosis of the painted lady butterfly *Vanessa cardui* (L.). Following this, Martín-Vega et al. [16] applied quantitative tomographic analyses to a morphological study of the development of the blow fly, *Calliphora vicina* Robineau-Desvoidy, providing volumetric and allometric data on selected organ systems during the intra-puparial period.

The aims of the current study are (i) to assess the potential of micro-CT as a tool of forensic relevance for a quantitative and qualitative analysis of the morphological changes taking place during the intra-puparial development of necrophagous blow flies and (ii) to develop a new, efficient and reliable method for assessing the insect age suitable for use by forensic entomology practitioners, combining both qualitative and quantitative measures of age.

## Material and methods

### Insect culture and sampling

Two blow fly species were selected for the current study: the common blue bottle blow fly *C. vicina* and the common green bottle blow fly *Lucilia sericata* (Meigen). These two species are among the most frequently used forensic indicators [8, 10, 11, 17–21, 24]. Laboratory colonies of *C. vicina* and *L. sericata* were established from adults collected using a modified Redtop^®^ fly trap (Miller Methods, Pretoria) in the Wildlife Garden of the Natural History Museum, London [26]. Newly emerged adults from the colonies were maintained at a controlled room temperature (23 ± 2 °C) and photoperiod (18:6 h, light:dark) to prevent the experimental population from entering diapause as post-feeding larvae [8, 16]. The flies were provided with sugar, milk powder and water ad libitum for 1 week. After the first week, they were also provided with 2 ml of pig blood (from pig liver) once daily during the following 10 days, as a protein uptake for egg maturation. The flies were subsequently starved for 4–5 days, in order to permit adequate time for egg development, after which fresh pig liver was provided as an oviposition medium.

Once the flies oviposited, the liver tissue with the eggs was transferred to a plastic box (160 mm × 160 mm × 86 mm) containing an approximately 3-cm layer of garden soil, sterilised by microwave to prevent the growth of microorganisms and placed into an incubator at one of three constant temperatures (15, 20 or 24 °C ± 0.8 °C) without light, following standard protocols for blow fly rearing [8, 11, 16, 24]. The larvae hatching from the eggs were allowed to feed and provided with more liver as needed. Once the post-feeding larvae started to wander, the box was checked every 6 h and the white prepupae, i.e. irreversibly contracted third instar larvae [13, 14], were placed into separate plastic boxes for each collection time (120 mm × 120 mm × 60 mm) containing an approximately 1.5-cm layer of autoclaved soil and labelled with the pupariation time. Collection times for blow fly puparia at each constant temperature were established using percentages of the total intra-puparial period, based on the data from unpublished studies carried out at the Natural History Museum (Richards et al., unpublished data). As mentioned above, correlating morphological landmarks to developmental percentages facilitates age estimation [20]. For *C. vicina*, ten puparia were collected at random at each of the 11 10% intervals of the total intra-puparial period, i.e. from 0% (corresponding to pupariation) to 100% (corresponding to adult emergence). For *L. sericata*, ten puparia were collected at random at 20, 40, 60, 80 and 100% of the total intra-puparial period. The collected puparia were killed and fixed in near-boiling water for ∼30 s and subsequently stored in 80% ethanol at 4 °C [27]. The entire procedure was replicated three times, using a different incubator for each constant temperature each time to avoid potential bias.

### Micro-CT scanning and analysis of tomographic data

Five random puparia from each batch of ten collected as described above were used for micro-CT scanning (i.e. 5 individuals × 3 replicates × 3 constant temperatures = 45 samples in total per each development interval). Each puparium was pierced in three places using an insect pin (in head, thoracic and abdominal segments) to enhance the penetration of the staining solution. Pierced puparia were stained by immersion in 0.5 M iodine in aqueous solution [8, 22] for 2 weeks and then washed and stored in 70% ethanol for 24 h before scanning. For scanning, each puparium was mounted in a plastic drinking straw containing 70% ethanol and sealed with plastic paraffin film (Parafilm M^®^; Bernis Company, Inc.). Each batch of five puparia from the same age and replicate was scanned together in a Nikon Metrology HMX ST 225 system (exposure 500 ms; voltage 110 kV; current 100 μA). The resulting projections were reconstructed with a voxel size of 9.5 μm in CT-Pro 2.1 (Nikon Metrology, Tring, UK).

For a qualitative analysis of the internal morphological changes, reconstructed slice stacks were rendered, reoriented and visualised for each scanned specimen using VG Studio Max 2.2 (Volume Graphics GmbH, Heidelberg, Germany). The contrast, grey value differences and voxel opacity were improved manually using the ‘volume rendering’ option. Virtual sections in the three principal planes (cross, horizontal and sagittal) were exported as JPEG stack files for each scanned specimen. Terminology for the different intra-puparial events and stages follows Fraenkel and Bhaskaran [13] and Martín-Vega et al. [14]. Terminology for the alimentary canal and the brain follows Graham-Smith [28] and Ito et al. [29], respectively.

**Fig. 1:**
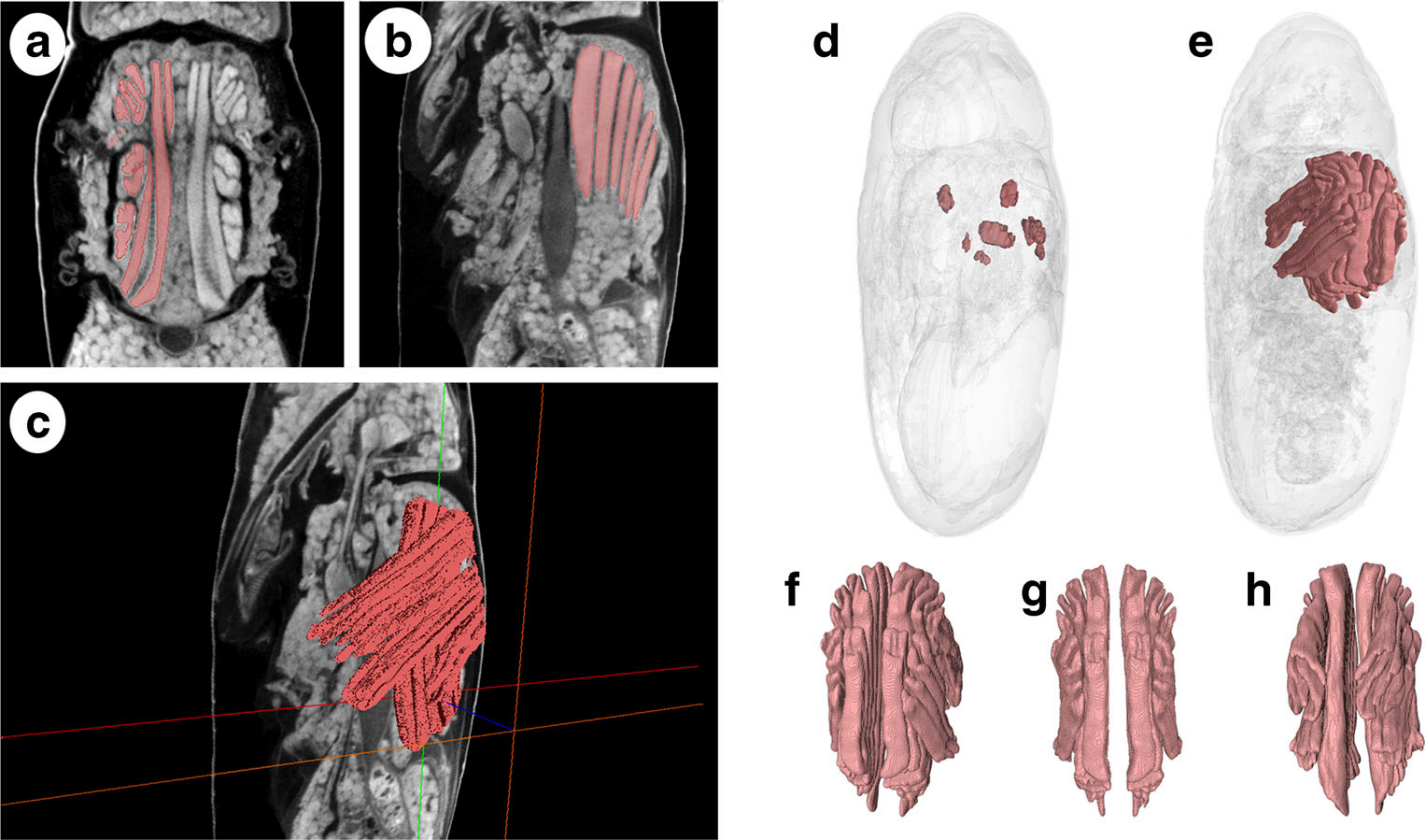
Method of segmentation for volume measurements and generation of 3D surface models. **a** Segmentation of the left set of indirect flight muscles (shown in *red*) in a micro-CT virtual horizontal section from a specimen at 70% of the total intra-puparial period (IPP). **b** Segmentation of the left set of indirect flight muscles (shown in *red*) in a micro-CT virtual sagittal section from a specimen at 70% IPP. **c** Volume rendering of the ongoing segmentation of the indirect flight muscles (shown in *red*) through several micro-CT virtual slices in a specimen at 70% IPP. **d** 3D surface model of a specimen at 20% IPP with the indirect flight muscles shown in *red*. **e** 3D surface model of a specimen at 100% IPP with the indirect flight muscles shown in *red*. **f** 3D surface model of the indirect flight muscles at 100% IPP, dorsolateral view. **g** 3D surface model of the indirect flight muscles at 100% IPP, dorsal view. **h** 3D surface model of the indirect flight muscles at 100% IPP, ventrolateral view

For a quantitative analysis, the stacks from five individuals of the 15 scanned at each temperature for each 10% development interval were randomly selected (i.e. 15 specimens per each 10% development interval were used in the quantitative analysis). The stacks from these individuals were loaded into Avizo 9.0 (Visualization Sciences Group, Bordeaux, France), and selected adult organ systems were segmented for volume measurements (Fig. 1). Two adult organ systems were selected for the quantitative analysis: the indirect flight muscles and the alimentary canal. The volumetric data from the 24 °C experimental set-up had been previously analysed [16], and both organ systems had been shown to change significantly in volume during the intra-puparial period. Here, we analyse triple the data, adding that from 15 and 20 °C. The indirect flight muscles are formed by two muscular sets, the dorso-longitudinal and dorso-ventral muscles [30], and their development was suggested as a potentially age-informative feature by Richards et al. [8]. The adult alimentary canal is the structure showing the most drastic changes in morphology during the intra-puparial period [16, 31]. None of these adult organ systems are present at the prepupal stage (0% of the total intra-puparial period), so those samples were excluded from the quantitative analysis. Also, in most puparia (37/45) collected at the 10% stage of the total intra-puparial development, the ‘explosion’ of the characteristic gas bubble formed at this stage (see “Results” for more details) precluded a quantitative analysis of those samples. Consequently, only those intact samples (8/45 in total) were segmented for volume measurements at the 10% stage of the total intra-puparial development.

Segmentation in Avizo 9.0 was performed automatically using the ‘Magic Wand’ tool after redefining the grey scale range for each particular region of interest for each specimen. The segmented volumes were then reviewed slice by slice and completed with manual segmentation where needed (Fig. 1). Quantitative data were calculated using the ‘material statistics’ module. As some sections from the foregut and the helicoidal region of the midgut were difficult to segment accurately due to lack of contrast, particularly in the last development intervals (70–100% of the total intra-puparial period), segmentation and data quantification of the alimentary canal were restricted to two different regions: the pre-helicoidal region of the midgut and the rectal pouch in the hindgut [16, 28]. Relative volumes of these two regions of the alimentary canal and of the indirect flight muscles were calculated as a percentage of the puparial volume. The use of relative volumes was preferred over actual volumes in order to minimise the potential effect of intra-specific variability in size among individuals of the same age.

The same segmentation and measurement procedure was replicated for *L. sericata* samples, but in this case, the stacks from five randomly selected individuals for each of the 20% development intervals from the three constant temperatures (i.e. five random samples in total for each development interval) were loaded into Avizo 9.0.

### Statistical analysis

Multi-factor analyses of variance (ANOVA) were performed to determine the statistical effect of the experimental rearing temperature and the percentage of the total intra-puparial development on the volume of each segmented organ in *C. vicina*. Differences were considered to be significant at the *p* < 0.05 level. When appropriate, differences were further investigated using one-way analysis of variance and post-hoc comparisons were carried out using the Bonferroni correction for multiple comparisons.

A linear discriminant analysis (LDA) was performed to develop a set of functions discriminating among the ten segmented development intervals (i.e. from 10 to 100% of the total intra-puparial period) for *C. vicina*. Those discriminant functions are linear combinations of continuous predictor variables, in this case the three volume measurements (the volume of the indirect flight muscles, the pre-helicoidal region of the midgut and the rectal pouch). To classify new cases, derived classification functions with the following formula were subsequently used to predict which developmental interval each sample belongs to

$$ {C}_j= k+\left({w}_1\times {X}_{j1}\right)+\left({w}_2\times {X}_{j2}\right)+\dots +\left({w}_p\times {X}_{j p}\right) $$

where *C*_*j*_ is the classification value for a sample *j*, *k* is the constant, *w* is the weight of the variable, *X*_*j*_ is the value of the variable for the sample *j* and *p* is the number of variables (3 in this study). A classification function was created for each of the ten development intervals, so any new sample *j* is classified as belonging to the development interval which shows the largest value of *C*_*j*_. The classification functions were tested with the samples used to fit the model (*N* = 143) and with 18 additional independent test samples. The test samples consisted of two puparia randomly collected among the remaining (i.e. not measured) puparia for each of the 20 to 100% development intervals (*N* = 18; not enough samples from the 10% percentage of the total intra-puparial development were available, see above). Test samples were segmented and measured as explained above. Moreover, the classification functions developed for *C. vicina* were also tested with the measured *L. sericata* puparia (*N* = 25), in order to see if a classifier developed for one species could be used for another at a family level. Statistical analyses were performed using Statgraphics Centurion (Statistical Graphics Corp. 1994–2000).

## Results

### Qualitative analysis of tomographic data

Seventeen age-informative internal morphological landmarks, easily identified in virtual micro-CT sections, were determined for *C. vicina* (Table 1). No differences in the relative timing of these qualitative landmarks were observed between the three temperature experimental set-ups or between males and females. These morphological landmarks allow for age estimation with a 10% temporal resolution from the 0 to 90% stages of the total intra-puparial period, although the distinctive characters between the 50 and 60% intervals must be considered with caution (see below). No clear morphological landmarks allowing for a distinction between the 90 and 100% time intervals could be found.

**Table 1:** Chronology of the development of the morphological landmarks diagnostic for the 11 10% time intervals of *Calliphora vicina* intra-puparial development, visible on virtual micro-CT sections

Morphological landmark	Temperature (°C)	0%	10%	20%	30%	40%	50%	60%	70%	80%	90%	100%
	15	154 ADD	179.2 ADD	204.4 ADD	229.6 ADD	254.8 ADD	280 ADD	305.2 ADD	330.4 ADD	355.6 ADD	380.8 ADD	406 ADD
	20	171 ADD	193.8 ADD	216.6 ADD	239.4 ADD	262.2 ADD	285 ADD	307.8 ADD	330.6 ADD	353.4 ADD	376.2 ADD	399 ADD
	24	161 ADD	184 ADD	207 ADD	230 ADD	253 ADD	276 ADD	299 ADD	322 ADD	345 ADD	368 ADD	391 ADD
Larval-pupal apolysis complete		−	+	+	+	+	+	+	+	+	+	+
Cephalopharyngeal skeleton within the insect body		Fully within	Partially within	−	−	−	−	−	−	−	−	−
Gas bubble		−	+	−	−	−	−	−	−	−	−	−
Head, wings and legs fully everted		−	−	+	+	+	+	+	+	+	+	+
Pupal-adult apolysis complete		−	−	−	+	+	+	+	+	+	+	+
Oesophagus fully developed		−	−	−	−	+	+	+	+	+	+	+
Position of crop		−	−	−	Anterior part of the thorax	Posterior part of the thorax	Abdomen	Abdomen	Abdomen	Abdomen	Abdomen	Abdomen
Shape of the adult midgut (in sagittal view)		−	Closed sack (displaced by gas bubble)	Closed sack (occupying the central body region)	Bottle	Long-necked bottle	Tube	Tube	Tube	Tube	Tube	Tube
Yellow body		−	+	+	+	+	+	+	+	+	−	−
Rectal pouch swollen (Fig. 4d–f)		−	−	−	−	−	−	−	−	+	+	+
Meconium in rectal pouch		−	−	−	−	−	−	−	−	± (96/4%)	+	+
Indirect flight muscles attached to adult cuticle		−	−	−	−	−	−	−	−	−	± (22/78%)	+
Position of thoracic ganglion		−	−	Anterior part of the thorax	1/3 of the thorax	1/3 of the thorax	1/3 of the thorax	1/3 of the thorax	1/3 of the thorax	1/3 of the thorax	1/3 of the thorax	1/3 of the thorax
Medulla and lobula visible		−	−	+	+	+	+	+	+	+	+	+
Shape of lamina (in horizontal view)		−	−	−	Bulge	Horseshoe	Horseshoe	Horseshoe or unfolded but not fully extended (13/87%)	Unfolded but not fully extended	Unfolded and fully extended	Unfolded and fully extended	Unfolded and fully extended
Lamina lying under the retina		−	−	−	−	−	−	−	+	+	+	+
Layer of crystal cones and primary pigment cells		−	−	−	−	−	−	−	−	−	+	+

The absence of a character is shown by a minus sign (−) and the presence by a plus sign (+). For those cases where variability was observed, the percentage of samples showing each option is given in brackets; otherwise, the character was present (or absent) in 100% of samples. Accumulated degree days (ADD) from egg laying have been calculated from the experimental cultures using a lower developmental threshold of 1 °C, as determined for *C. vicina* in London, UK [11]

Overall, sagittal sections were found to be the most suitable to visualise those morphological landmarks related to the completion of the apolysis events, the head eversion and the development of the alimentary canal (Table 1). On the other hand, horizontal sections were found to be the most suitable to visualise the morphological landmarks related to the development of the brain and the eyes (Table 1). Consequently, it is important to emphasise that one of the main advantages of micro-CT is the possibility of virtually dissecting the same specimen in any plane, yielding complementary views of the same structure when needed.

**Fig. 2:**
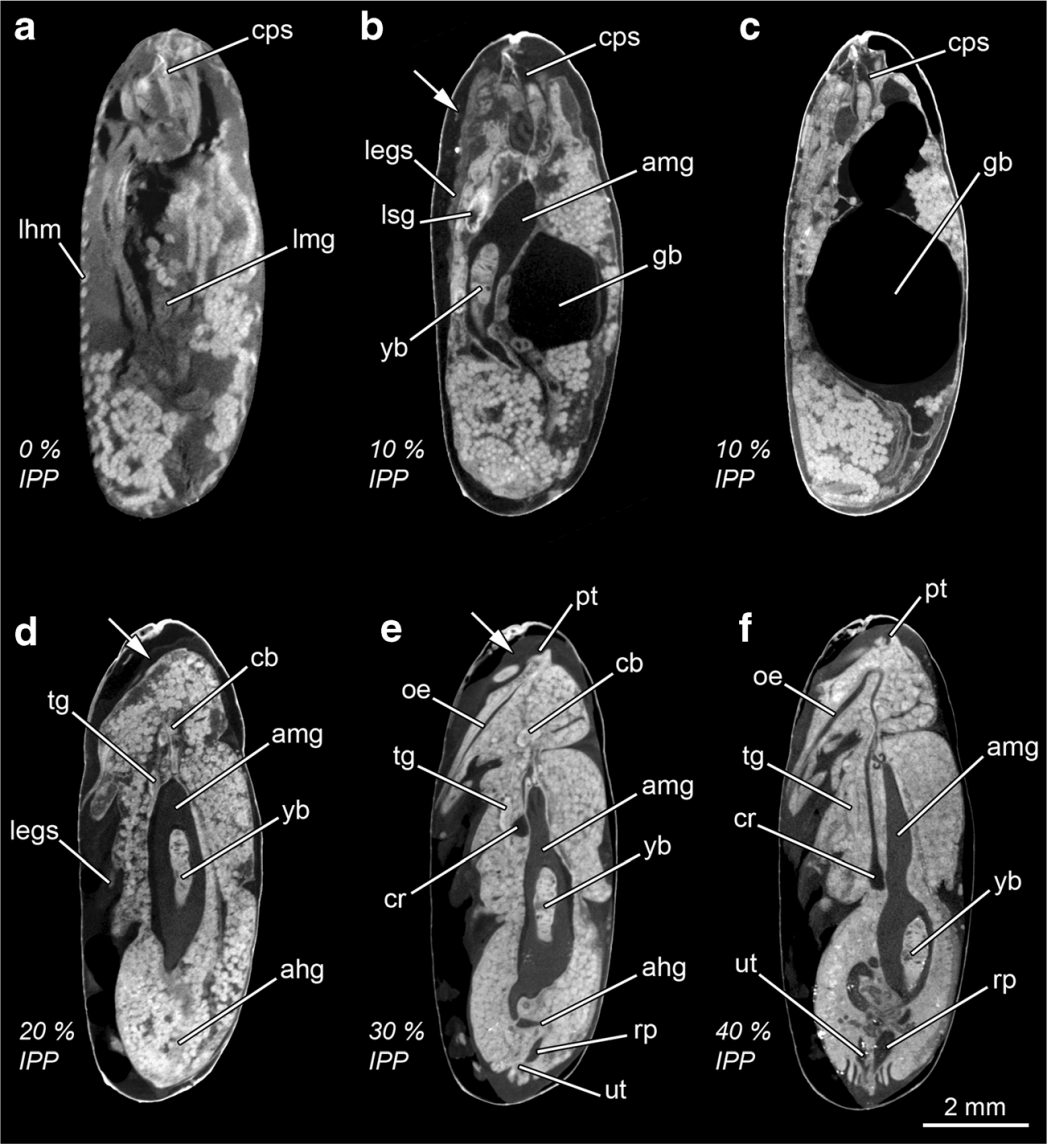
Micro-CT-based virtual medial sagittal sections of *Calliphora vicina* puparia at different percentages of time of the total intra-puparial period (*IPP*). **a** 0% IPP. **b** 10% IPP. *Arrow* shows the separation of the pupal epidermis from the larval cuticle (i.e. puparium). **c** Specimen at 10% IPP where the gas bubble (*gb*) exploded during hot water fixation. **d** 20% IPP. *Arrow* shows the separation of the adult epidermis from the pupal cuticle; note that the separation is not complete over all the body. **e** 30% IPP. *Arrow* shows the separation of the adult epidermis from the pupal cuticle; note that the separation is now complete over all the body. **f** 40% IPP. *ahg* adult hindgut, *amg* adult midgut, *cb* central brain, *cps* cephalopharyngeal skeleton, *cr* crop, *gb* gas bubble, *legs* legs, *lhm* larval hypodermal muscles, *lmg* larval midgut, *lsg* larval salivary glands, *oe* oesophagus, *pt* ptilinum, *rp* rectal pouch, *tg* thoracic ganglion, *ut* uterus, *yb* yellow body

**Fig. 3:**
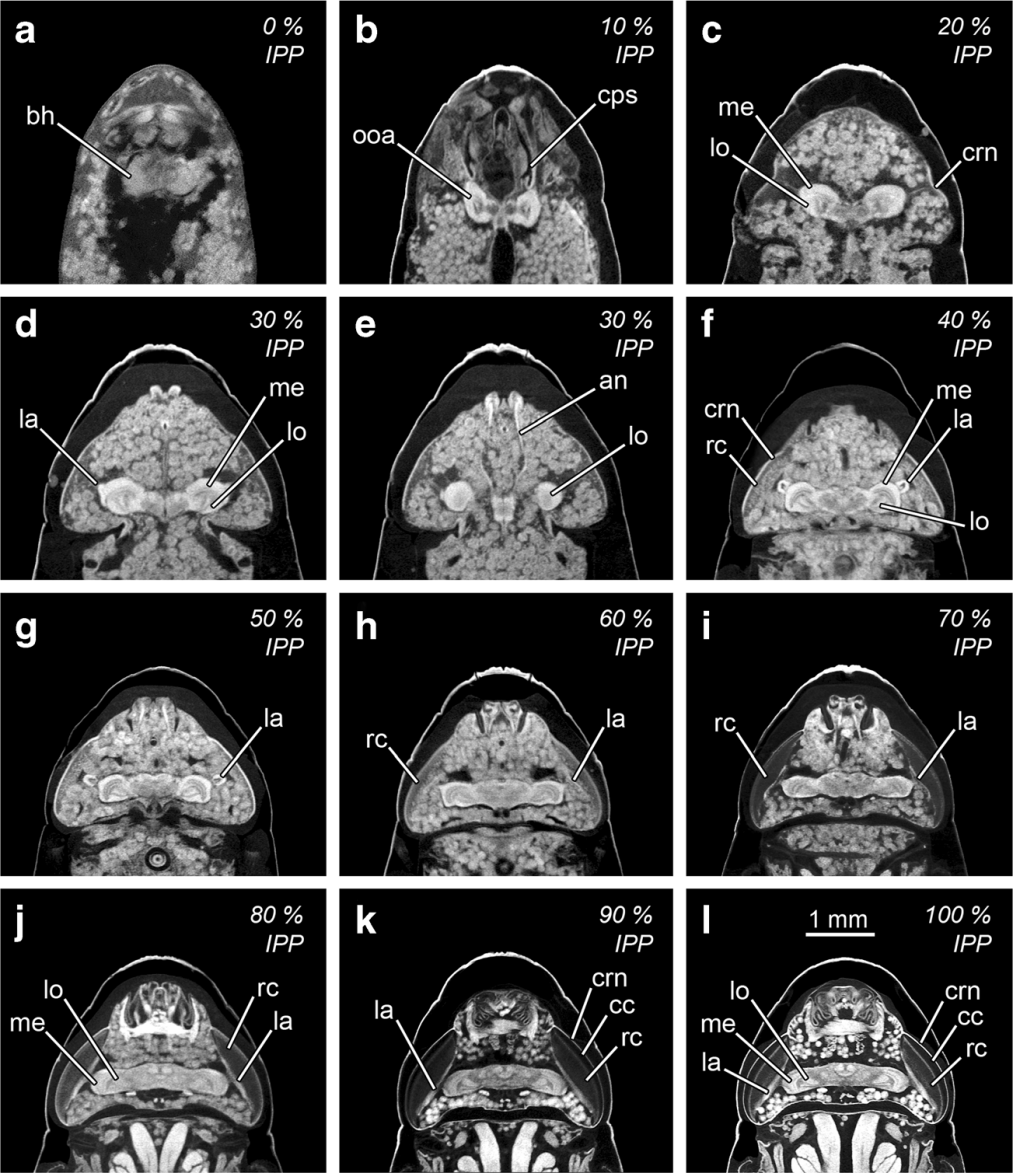
Micro-CT-based virtual medial horizontal sections of the head and anterior thoracic regions of *Calliphora vicina* puparia at different percentages of time of the total intra-puparial period (*IPP*). **a** 0% IPP. **b** 10% IPP. **c** 20% IPP. **d** 30% IPP. **e** 30% IPP, ventral medial section. **f** 40% IPP. **g** 50% IPP. **h** 60% IPP. **i** 70% IPP. **j** 80% IPP. **k** 90% IPP. **l** 100% IPP. *an* antennal nerve, *bh* brain hemispheres, *cc* crystal cones, *cps* cephalopharyngeal skeleton, *crn* cornea, *la* lamina, *lo* lobula, *me* medulla, *ooa* outer optic lobe anlagen, *rc* retinular cells

**Fig. 4:**
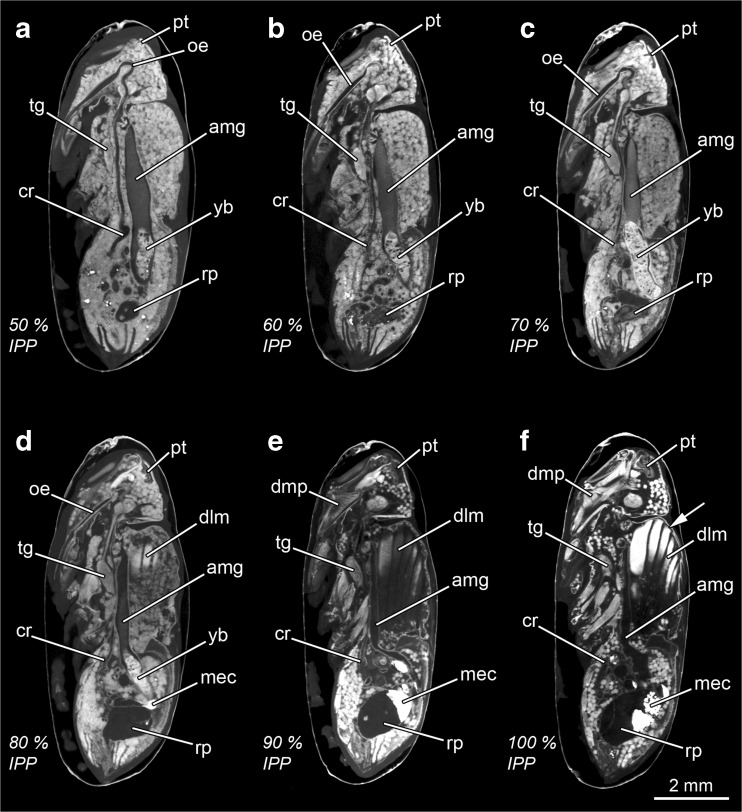
Micro-CT-based virtual medial sagittal sections of *Calliphora vicina* puparia at different percentages of time of the total intra-puparial period (*IPP*). **a** 50% IPP. **b** 60% IPP. **c** 70% IPP. **d** 80% IPP. **e** 90% IPP. **f** 100% IPP, slightly left lateral-medial sagittal section to show the indirect flight muscles attached to the adult cuticle (*arrow*). *amg* adult midgut, *cr* crop, *dlm* dorsal longitudinal muscles, *dmp* dilator muscle of the pharynx, *mec* meconium, *oe* oesophagus, *pt* ptilinum, *rp* rectal pouch, *tg* thoracic ganglion, *yb* yellow body

**Fig. 5:**
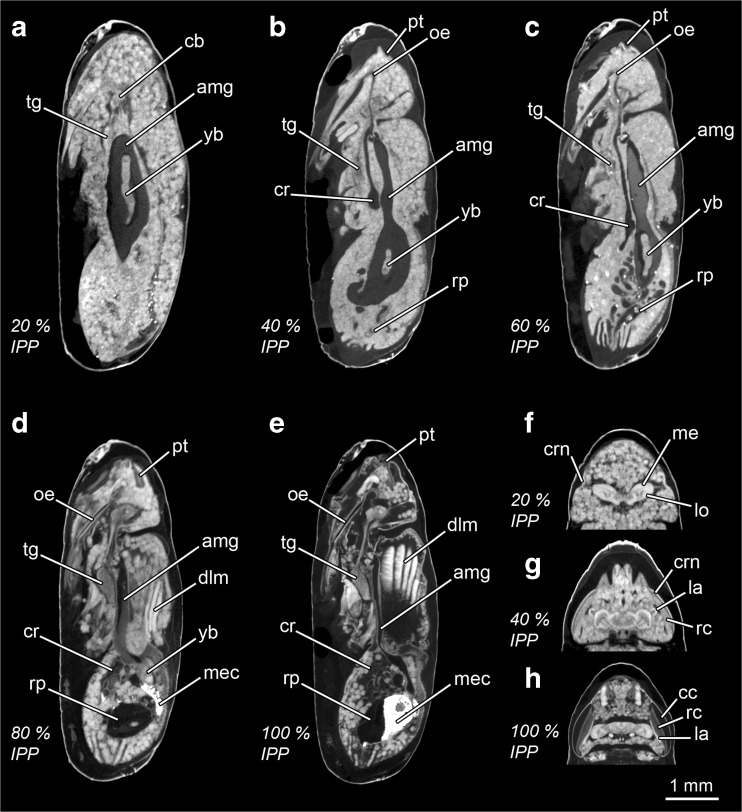
Micro-CT-based virtual sections of *Lucilia sericata* puparia at different percentages of time of the total intra-puparial period (*IPP*). **a** 20% IPP, medial sagittal section. **b** 40% IPP, medial sagittal section. **c** 60% IPP, medial sagittal section. **d** 80% IPP, medial sagittal section. **e** 100% IPP, medial sagittal section. **f** head at 20% IPP, medial horizontal section. **g** head at 40% IPP, medial horizontal section. **h** head at 100% IPP, medial horizontal section. *amg* adult midgut, *cb* central brain, *cc* crystal cones, *cr* crop, *crn* cornea, *dlm* dorsal longitudinal muscles, *la* lamina, *lo* lobula, *me* medulla, *mec* meconium, *oe* oesophagus, *pt* ptilinum, *rc* retinular cells, *rp* rectal pouch, *tg* thoracic ganglion, *yb* yellow body

It is not our aim here to provide a detailed account of the internal morphological changes and processes taking place during the intra-puparial period of *C. vicina*. Such detailed morphological descriptions and definitions of terms and concepts can be found elsewhere [14–16]. Here, only the morphological characters, which are age informative (Table 1), are labelled in Figs. 2, 3, 4 and 5. Nevertheless, the main qualitative changes related to the age-informative morphological landmarks throughout development (Table 1) are summarised below, and some related concepts are briefly described. It is worth mentioning that the three developmental stages taking place inside the puparium (prepupa, pupa and pharate adult) are delimited by the completion of the larval-pupal and pupal-adult apolysis, respectively [14]. An apolysis is defined as the separation of the epidermal cells from the cuticle. Hence, the larval-pupal apolysis is the separation of the pupal epidermal cells from the larval cuticle (i.e. the puparium) and the pupal-adult apolysis is the separation of the adult epidermal cells from the pupal cuticle.

#### *C. vicina*

##### 0% of the total intra-puparial period (Figs. 2a and 3a)

Just after pupariation, the internal morphology is clearly distinct from the morphology shown during the remaining intra-puparial period (Fig. 2a). The internal morphology at this stage corresponds to a contracted post-feeding larva, and accordingly, the cephalopharyngeal skeleton can be visualised embedded in the anterior body region. The majority of the body is occupied by the coiled, tubular larval midgut, which is emptied before pupariation in cyclorrhaphous flies [13]. At this time, the insect should be called a prepupa [13, 14], as the internal tissues are still attached to the puparium (i.e. the larval cuticle) (Table 1), with the larval hypodermal musculature easily distinguishable (Fig. 2a). The brain shows the typical anatomy described for cyclorrhaphous fly larvae [31, 32], with two large brain hemispheres (Fig. 3a).

##### 10% of the total intra-puparial period (Figs. 2b, c and 3b)

At this development interval, the insect is a pupa, as the larval-pupal apolysis is complete (i.e. the epidermis has separated from the larval cuticle or puparium all along the insect body) (Fig. 2b). This stage is called the cryptocephalic (‘hidden head’) pupa [14] as the wings and legs are partially everted, but the head has not yet everted (Table 1). The cephalopharyngeal skeleton has been partially extruded, but the dorsal and ventral cornua are still embedded in the thorax (Fig. 2b). The apoptotic larval salivary glands appear as very bright structures extending through the ventral side of the thorax and abdominal regions. Nevertheless, the most distinct morphological landmark of this development interval is the presence of a large gas bubble within the apoptotic larval tissues in the abdominal region (Table 1, Fig. 2b). The gas bubble plays an essential role in the later eversion of the head and is thought to keep the body volume constant within the rigid puparium during the period of extensive apoptosis of the larval tissues [15]. The adult midgut is already present in this development interval in the form of a closed sack containing the yellow body, i.e. the mass of apoptotic larval midgut cells (Fig. 2b). The adult midgut is displaced to the ventral side by the gas bubble, acquiring a typical curved shape (Fig. 2b). The outer optic lobe anlagen are visualised as two symmetrical, very bright C-shaped structures, one on each brain hemisphere (Fig. 3b). The outer optic lobe anlagen are layers composed of neuroblasts which, together with the neuroblasts from the inner optic lobe anlagen (not distinguishable), divide, forming the optic lobes [33]. It must be mentioned that in most puparia (37/45) collected at the 10% stage of the total intra-puparial development, the micro-CT scans revealed that the gas bubble had apparently ‘exploded’. This hot water-induced explosion of the gas bubble during killing and fixation displaced and destroyed the adjacent pupal tissues (Fig. 2c), thus precluding both qualitative and quantitative morphological analyses of those samples.

##### 20% of the total intra-puparial period (Figs. 2d and 3c)

Full eversion of head, legs and wings takes place between 10 and 20% of the total intra-puparial period. With the head eversion, the cryptocephalic pupa transforms into a phanerocephalic pupa (‘visible head’) [13, 14]. The insect is still a pupa at 20% of the total intra-puparial period, because although pupal-adult apolysis (i.e. the separation of the epidermis from the pupal cuticle) has started, it is not complete over the entire body (Table 1, Fig. 2d). The shape of the adult midgut is probably the most distinctive morphological character at this developmental stage (Table 1). The gas bubble disappears during head eversion (see [15] for a detailed account of this process) so the sack-shaped adult midgut, still closed at both ends, expands to occupy its central position within the internal tissues of the pupa (Fig. 2d). The adult hindgut starts to proliferate, and a minute rectal pouch can be observed in some specimens only, so this character should be treated with caution as an age indicator and, therefore, it is not included in Table 1. Most adult structures appear at this stage and will develop progressively during the subsequent development intervals, like the antennae, the indirect flight muscles and the adult salivary glands. The apoptotic larval salivary glands (much reduced in length compared to the previous development interval, cf. Fig. 2b) are located at the anterior end of the thorax, although they are not always clearly observable and, therefore, they are omitted here (see [16] for more details of this structure). Nevertheless, the neck is not fully developed so that the boundary between head and thorax is vague. The thoracic ganglion appears at this stage and is located close to the apoptotic larval salivary glands (Fig. 2d). It must be mentioned that small and short fibres of the indirect flight muscles are discernible at this stage in some sections of the thorax (but not visible in Fig. 2d). These muscular fibres will develop progressively during the subsequent development intervals; more details on the morphological development of the indirect flight musculature can be found in [16]. In the head, the cornea is clearly visible, although no retinular cells are discernible (Fig. 3c). In the brain, the two optic lobes are already present and clearly distinguishable at this stage. The optic lobe is a highly retinotopically ordered neuropil—i.e. a synapse-rich region where neuronal processes contact and form synaptic connections [29]—that consists of three smaller and distinguishable neuropils: the lamina, the medulla and the lobula complex [33, 34]. At 20% of the total intra-puparial period, the medulla and lobula cortices are clearly identifiable but the lamina is still not clearly discernible (Fig. 3c).

##### 30% of the total intra-puparial period (Figs. 2e and 3d, e)

At the 30% stage of the total intra-puparial period, pupal-adult apolysis is complete over all the body (Table 1; Fig. 2e); hence, the insect should no longer be called a pupa but, instead, a pharate adult [13, 14]. The apoptotic larval salivary glands are no longer observable. The ptilinal invagination is present at the front of the head and will develop progressively during subsequent development intervals [16]. The shape of the adult midgut changes distinctly from the large, ovoid sack characteristic of the previous development interval to a bottle- or wineskin-shaped sack, with the anterior portion narrowed and stretched (Table 1; Fig. 2e). The adult foregut is discernible, although the oesophagus is not fully developed and the crop appears as a ball-like structure located in the anterior region of the thorax, ventrally to the stretched region of the adult midgut. The adult hindgut is a continuous tube with a clearly discernible (although small) rectal pouch (Fig. 2e). The gonads and reproductive organs are also fully discernible at this development interval, with no difference in the time of appearance between males and females. In the brain, the medulla and lobula cortices develop further and the lamina starts to be discernible as a bulge dorsal to the medulla (Fig. 3d). A thin layer of retinular cells is present in the eye at this stage, but surrounded by adipose and connective tissue and not connected to the lamina. The antennal nerves also appear at this stage (Fig. 2e).

##### 40% of the total intra-puparial period (Figs. 2f and 3f)

In the foregut, the oesophagus is fully developed, whereas the crop duct lengthens and, as a result, the crop is now located in the posterior region of the thorax (Table 1; Fig. 2f). The final portion of the adult midgut becomes helicoidal in the abdomen. The pre-helicoidal region of the adult midgut acquires the shape of a long-necked bottle, with the thoracic part stretched and narrower than the abdominal part which is expanded and wider. The cervical connective nerve, which connects the gnathal and the thoracic ganglia, lengthens, and the thoracic ganglion reaches its final position in approximately the centre of the ventral region of the thorax. In the brain, the lamina bends longitudinally along the dorsal/ventral body axis forming a semicircle or ‘horseshoe’ in horizontal section (Fig. 3f), with the tips of the semicircle directed towards the medulla. In the eye, the layer of retinular cells develops further but it is not yet connected to the lamina.

##### 50% of the total intra-puparial period (Figs. 3g and 4a)

The crop duct lengthens further, and the crop reaches its final position in the anterior portion of the abdomen, where it expands and flattens (Fig. 4a). The pre-helicoidal region of the midgut becomes cylindrical or tube shaped, with no distinctively stretched or expanded areas in sagittal section. The brain and the eyes show no significant changes compared to the 40% stage of the total intra-puparial period (Fig. 3g).

##### 60% of the total intra-puparial period (Figs. 3h and 4b)

The foregut, midgut and hindgut show no significant new qualitative changes in morphology (Fig. 4b). The lamina unfolds and acquires a flattened shape, extended parallel to the layer of retinular cells of the eye in horizontal section; however, there is still adipose and connective tissue between the retina and the lamina (Fig. 3h). Moreover, the length of the lamina in horizontal view is very short, only equal to a small portion of the layer of retinular cells. It must be mentioned that some 60% specimens (4/15, 0/15 and 2/15 from the 15, 20 and 24 °C experimental set-ups, respectively) showed a horseshoe-shaped lamina in horizontal view, as characteristic of the 40–50% periods of the total intra-puparial development, but all showed a tube-shaped pre-helicoidal region of the midgut, as characteristic of the 50–80% periods of the total intra-puparial development.

##### 70% of the total intra-puparial period (Figs. 3i and 4c)

The foregut, midgut and hindgut again show no significant new qualitative changes in morphology (Fig. 4c). In the eye, the retina becomes much thicker and homogeneously darker as the retinular cells differentiate (Fig. 3i). The lamina extends further, lying under the retina.

##### 80% of the total intra-puparial period (Figs. 3j and 4d)

The indirect flight muscles are clearly more developed with thicker fibres compared to previous development intervals, although fat bodies and undifferentiated tissue are still present between the two sets of longitudinal muscles in the medial sagittal section (Fig. 4d). Moreover, they are not yet attached to the adult cuticle and appear surrounded by fat bodies. The rectal pouch increases significantly in volume, with a clearly swollen appearance compared to previous development intervals (Fig. 4d). The yellow body is still present, and the meconium, i.e. the waste products of metamorphosis [35], can be distinguished as a very bright body (Fig. 4d). The meconium starts to fill the rectum and, in some specimens (2/15 from the 24 °C experimental set-up), also a small portion of the rectal pouch (Table 1). In the eye, the lamina reaches its final extension, lying under and along the retina (Fig. 3j).

##### 90–100% of the intra-puparial development (Figs. 3k, l and 4e, f)

The indirect flight muscles are fully developed and attached to their corresponding insertion points in the thoracic cuticle (Fig. 4f). This character is not always clear at 90% of the total intra-puparial period, as some fat bodies can be observed between the muscles and the cuticle in some sections (Table 1). Also, at 90% of the total intra-puparial period, some specimens (9/15, 1/15 and 0/15 from the 15, 20 and 24 °C experimental set-ups, respectively) still showed a variable amount of fat bodies and undifferentiated tissue between the two sets of longitudinal indirect flight muscles in the medial sagittal section. However, the remaining individuals scanned at 90% of the total intra-puparial period, and all the specimens scanned at 100%, showed no tissue between the two sets of longitudinal indirect flight muscles in the medial sagittal section (Fig. 4e, f).The pre-helicoidal region of the midgut narrows to its minimum diameter, and the yellow body is completely absorbed, i.e. no longer observable (Fig. 4e, f). The rectal pouch increases in volume, and it is partially filled with meconium. In the head, the dilator muscle of the pharynx and the ptilinal invagination are fully developed. The eyes are also fully developed, showing two distinct cell layers between the cornea and the lamina; the layer lying under the cornea is formed by crystalline cones surrounded by primary pigment cells, and the layer lying above the lamina is formed by the retinular cells (Fig. 3k, l). Overall, no clear qualitative differences allowing for discrimination between the 90 and the 100% stages of the total intra-puparial period could be determined.

#### *L. sericata*

Finally, the specimens of *L. sericata* scanned at 20, 40, 60, 80 and 100% of the total intra-puparial period (Fig. 5) showed the same morphological markers as those determined at the same developmental intervals for *C. vicina* (Table 1). Similar to *C. vicina*, some specimens (6/15, 2/15 and 8/15 from the 15, 20 and 24 °C experimental set-ups, respectively) at 60% of the total intra-puparial period showed a horseshoe-shaped lamina in horizontal view, as characteristic of the 40–50% of the total intra-puparial development, but a tube-shaped pre-helicoidal region of the midgut, as characteristic of the 50–80% of the total intra-puparial development (Fig. 5c–g).

### Quantitative analysis of tomographic data

#### *C. vicina*

**Fig. 6:**
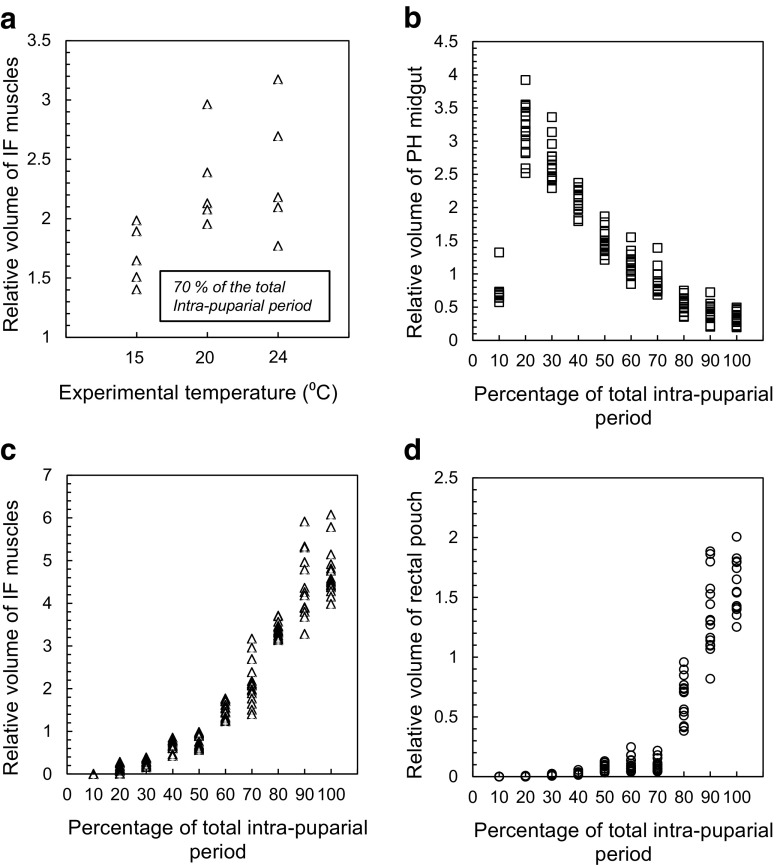
Relative volumes of segmented structures in *Calliphora vicina* puparia: indirect flight muscles (triangles), pre-helicoidal region of the midgut (squares) and rectal pouch (circles). **a** Indirect flight (*IF*) muscles at 70% of the total intra-puparial period. **b** Pre-helicoidal (*PH*) region of the midgut during the entire intra-puparial period. **c** IF muscles during the entire intra-puparial period. **d** Rectal pouch during the entire intra-puparial period

As expected, the total period of intra-puparial development, measured at 10% intervals, had a significant effect on the volume of the three segmented structures in *C. vicina* (indirect flight muscles: *F* = 363.29, *p* < 0.05; pre-helicoidal region of the midgut: *F* = 317.1, *p* < 0.05; rectal pouch: *F* = 265.67, *p* < 0.05). However, the experimental temperature only had a significant effect on the volume of the indirect flight muscles (*F* = 5.82, *p* < 0.05), but not on the other two structures (pre-helicoidal region of the midgut: *F* = 0.5, *p* > 0.05; rectal pouch: *F* = 0.47, *p* > 0.05). Nevertheless, within the volume of the indirect flight muscles, significant differences among the experimental rearing temperatures were only found at 70% of the total intra-puparial period (*F* = 5.14, *p* < 0.05). Pairwise comparisons between the average ranks using the Bonferroni procedure only showed significant differences between the volume data from the 15 and 24 °C experimental temperature set-ups at 70% of the total intra-puparial period. At this time interval, the mean volume of the indirect flight muscles was lower in the 15 °C temperature set-up in comparison to the other temperatures, although the ranges of the volume values overlapped among the three experimental temperatures (Fig. 6a).

Overall, in *C. vicina*, the pre-helicoidal region of the midgut shows a clear increase in the volume between the 10 and 20% stages of the total intra-puparial development (Fig. 6b). This increase coincides with the major morphological transformation of the cryptocephalic pupa into the phanerocephalic pupa and the displacement of the gas bubble, so the adult midgut increases to occupy the central region of the insect body (Fig. 2b–d). However, from the 20% time interval to the end of the intra-puparial period, the pre-helicoidal region of the midgut slowly and progressively decreases in relative volume (Fig. 6b) as the midgut becomes more tubular and the indirect flight muscles progressively occupy the majority of the thorax (Figs. 2d–f and 3). Consequently, the changes in the volume of the indirect flight muscles follow a clearly opposite pattern in comparison to the pre-helicoidal volume (Fig. 6c). However, the increase in volume of the indirect flight muscles is not as gradual as the decrease of the volume of the pre-helicoidal region of the midgut: there appears to be a short burst in growth followed by a period of relative stasis at 40% and then another burst at 60% of the total intra-puparial development (Fig. 6c). The 70% time interval, more accurately the 60–80% interval, appears to include a major increase in muscle volume focussed within a relatively short period as it showed a particularly wide range in the values of indirect flight muscles volume (Fig. 6c; Table 2). As mentioned, 70% was the time interval where significant differences between the three experimental temperatures were found. Wide ranges in the values of the indirect flight muscles were observed at 20 and 24 °C, but not at 15 °C (Fig. 6a). At 80 and 90% time intervals, the volume of the indirect flight muscles shows another growth burst before achieving its final volume at 90–100% of the total intra-puparial development (Fig. 6c). Finally, the rectal pouch also increases in volume during the intra-puparial period, although the increase during the first 70% of the total period is virtually negligible and it only shows a single phase of rapid increase focussed between 70 and 90% of the total period, respectively (Fig. 6d). Nevertheless, the values of the volumes of the three measured systems frequently overlapped among consecutive intervals (Fig. 6b–d).

**Table 2:** Volume measurements of the pre-helicoidal midgut, the indirect flight muscles and the rectal pouch at 10% intervals from pupariation to adult emergence in *Calliphora vicina*

Developmental interval (as a percentage of the total intra-puparial period)	Relative volume of pre-helicoidal midgut (average ± STD)	Relative volume of pre-helicoidal midgut (range)	Relative volume of indirect flight muscles (average ± STD)	Relative volume of indirect flight muscles (range)	Relative volume of rectal pouch (average ± STD)	Relative volume of rectal pouch (range)
0	0	0	0	0	0	0
10	0.74 ± 0.3	0.57–1.32	0	0	0	0
20	3.17 ± 0.38	2.51–3.91	0.12 ± 0.09	0.005–0.28	0.001 ± 0.002	0–0.008
30	2.66 ± 0.29	2.29–3.35	0.27 ± 0.07	0.15–0.38	0.01 ± 0.005	0.005–0.02
40	2.1 ± 0.17	1.79–2.37	0.64 ± 0.12	0.42–0.85	0.02 ± 0.01	0.01–0.05
50	1.47 ± 0.17	1.21–1.86	0.75 ± 0.15	0.56–0.98	0.07 ± 0.03	0.03–0.12
60	1.14 ± 0.17	0.84–1.55	1.48 ± 0.19	1.23–1.77	0.1 ± 0.06	0.03–0.24
70	0.88 ± 0.18	0.68–1.39	2.12 ± 0.5	1.4–3.17	0.09 ± 0.05	0.04–0.21
80	0.53 ± 0.11	0.35–0.74	3.37 ± 0.18	3.13–3.7	0.64 ± 0.18	0.38–0.95
90	0.41 ± 0.12	0.2–0.72	4.36 ± 0.73	3.28–5.91	1.35 ± 0.31	0.81–1.88
100	0.34 ± 0.09	0.19–0.49	4.71 ± 0.57	3.98–6.07	1.59 ± 0.22	1.25–2

Relative volumes are given as a percentage of the total puparial volume

**Fig. 7:**
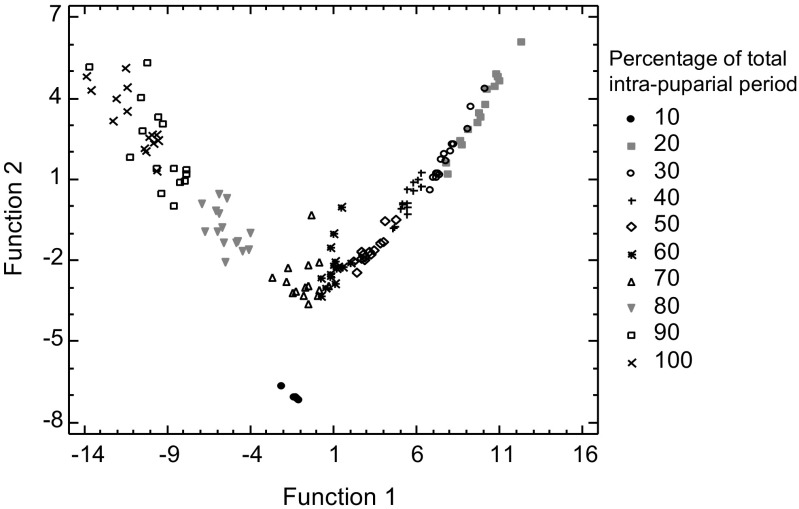
Plot of the first two discriminant functions for the 10% intervals of the intra-puparial period of *Calliphora vicina*

The three quantitative variables were incorporated into a LDA model to discriminate between the ten percentage-based divisions of the total intra-puparial development of *C. vicina*, i.e. from 10 to 100%. The model contained three discriminant functions with the first two functions accounting for the highest percentage of variance in the datasets (84.56 and 13.96% of the variance, respectively) (Table 3; Fig. 7). Overall, the first two discriminant functions separated the groups moderately well, but leaving some overlap between adjacent ones; the overlap was particularly high between the 20 and the 30% and between the 90 and the 100% development intervals (Fig. 7). On the other hand, the 10% development interval group was clearly separated from the rest (Fig. 7). Table 4 shows the functions used to classify observations. Validation with the samples used to fit the model gave a percentage of cases correctly classified (i.e. specimens correctly assigned to their actual age) of 86.01% (Table 5). In all the cases incorrectly classified, the correct group scored the second highest with similar values to the highest value (data not shown). Most misclassifications were found between the 20 and 30% and between the 90 and 100% of the total intra-puparial development, reflecting the overlap already commented on, with some additional misclassifications of individuals at the 50 and 70% of the total intra-puparial period misclassified as 40 and 60%, respectively (Table 5). On the other hand, validation with the independent test samples classified 94.44% of cases correctly, with only one sample at the 50% interval of the total intra-puparial development misclassified as belonging to the 40% interval (Table 6).

**Table 3:** Standardised discriminant function coefficients for the intra-puparial development intervals of *Calliphora vicina*

	Function 1	Function 2	Function 3
Relative volume of indirect flight muscles	−0.523611	0.245734	0.846463
Relative volume of the pre-helicoidal region of the midgut	0.664104	0.713722	0.232452
Relative volume of rectal pouch	−0.459161	0.567399	−0.722694
Relative percentage	84.56	13.96	1.48

**Table 4:** Classification function coefficients for the percentage of the total intra-puparial development of *Calliphora vicina*

	10%	20%	30%	40%	50%	60%	70%	80%	90%	100%
Constant (*k*)	−4.4774	−122.495	−87.4043	−56.7207	−30.3945	−26.354	−28.7956	−50.9559	−99.506	−122.636
Weight (*w*) of the variable										
Indirect flight muscles	5.80513	1.98545	2.91656	5.57557	5.97602	11.3821	16.3072	23.4428	27.9186	29.5502
Pre-helicoidal region of the midgut	0.241616	75.5737	63.504	50.1271	35.1466	27.546	21.4719	12.3071	8.09613	5.92862
Rectal pouch	−3.32955	−7.8996	−6.77473	−6.37318	−2.63901	−3.8278	−6.42504	17.8515	50.9803	62.1799

**Table 5:** Classification matrix for the 143 samples used to fit the model to discriminate among the ten percentages of the total intra-puparial development of *Calliphora vicina*, i.e. from 10 to 100%

Percentage	10%	20%	30%	40%	50%	60%	70%	80%	90%	100%	Percentage correctly classified
10	**8**	0	0	0	0	0	0	0	0	0	100
20	0	**11**	*4*	0	0	0	0	0	0	0	73.33
30	0	*3*	**11**	*1*	0	0	0	0	0	0	73.33
40	0	0	0	**15**	0	0	0	0	0	0	100
50	0	0	0	*1*	**14**	0	0	0	0	0	93.33
60	0	0	0	0	0	**15**	0	0	0	0	100
70	0	0	0	0	0	*2*	**13**	0	0	0	86.66
80	0	0	0	0	0	0	0	**15**	0	0	100
90	0	0	0	0	0	0	0	0	**12**	*3*	80
100	0	0	0	0	0	0	0	0	*6*	**9**	60
Total	8	14	15	17	14	17	13	15	18	12	**86.01**

Column headings correspond to the estimated percentages and row headings to the actual percentages. Values showing correct classifications are presented in bold, and those showing misclassifications are presented in italics

**Table 6:** Classification matrix for the 18 test samples of *Calliphora vicina*

Percentage	10%	20%	30%	40%	50%	60%	70%	80%	90%	100%	Percentage correctly classified
20	0	**2**	0	0	0	0	0	0	0	0	100
30	0	0	**2**	0	0	0	0	0	0	0	100
40	0	0	0	**2**	0	0	0	0	0	0	100
50	0	0	0	*1*	**1**	0	0	0	0	0	50
60	0	0	0	0	0	**2**	0	0	0	0	100
70	0	0	0	0	0	0	**2**	0	0	0	100
80	0	0	0	0	0	0	0	**2**	0	0	100
90	0	0	0	0	0	0	0	0	**2**	0	100
100	0	0	0	0	0	0	0	0	0	**2**	100
Total	0	2	2	3	1	2	2	2	2	2	**94.44**

Column headings correspond to the estimated percentages and row headings to the actual percentages. Values showing correct classifications are presented in bold, and those showing misclassifications are presented in italics

#### *L. sericata*

**Fig. 8:**
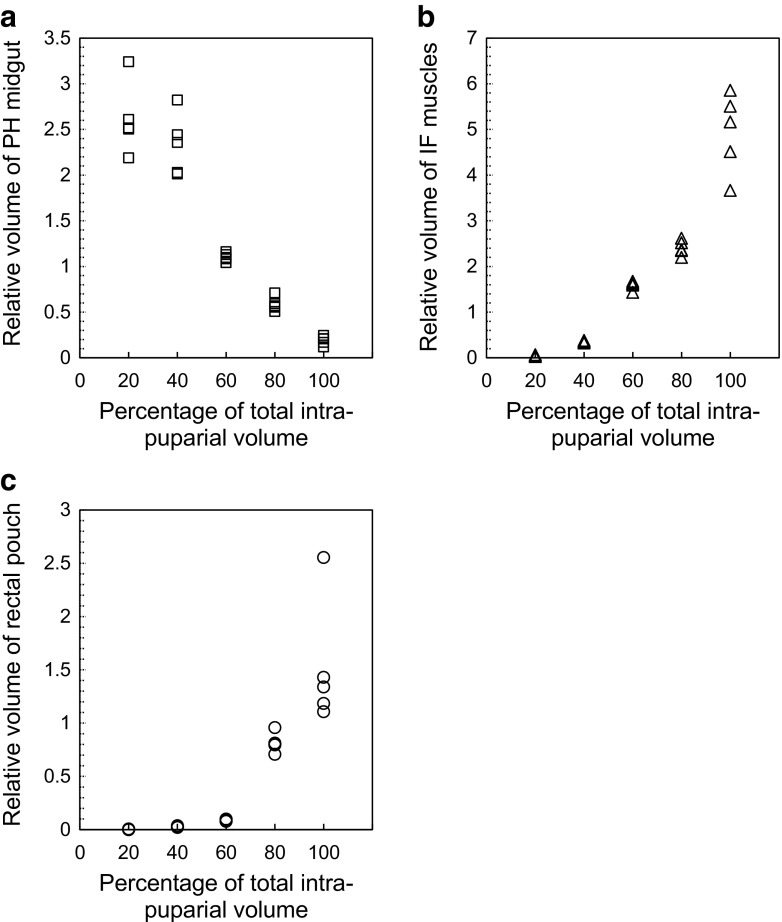
Relative volumes of segmented structures during the entire intra-puparial period of *Lucilia sericata*: indirect flight muscles (triangles), pre-helicoidal region of the midgut (squares) and rectal pouch (circles). **a** Pre-helicoidal region of the midgut. **b** Indirect flight muscles. **c** Rectal pouch

The volume measurements of the pre-helicoidal midgut, the indirect flight muscles and the rectal pouch in *L. sericata* showed similar values and a similar progression through development when compared to *C. vicina* (Fig. 8; Table 7). However, the LDA model developed for *C. vicina* showed a poor performance when validated with the *L. sericata* samples, with a percentage of cases correctly classified of 68% (Table 8).

**Table 7:** Volume measurements of the pre-helicoidal midgut, the indirect flight muscles and the rectal pouch at different times from pupariation to adult emergence in *Lucilia sericata*

Developmental interval (as a percentage of the total intra-puparial period)	Relative volume of pre-helicoidal midgut (average ± STD)	Relative volume of pre-helicoidal midgut (range)	Relative volume of indirect flight muscles (average ± STD)	Relative volume of indirect flight muscles (range)	Relative volume of rectal pouch (average ± STD)	Relative volume of rectal pouch (range)
20	2.61 ± 0.38	2.18–3.24	0.04 ± 0.01	0.02–0.06	0.002 ± 0.002	0–0.006
40	2.33 ± 0.33	2.01–2.82	0.35 ± 0.02	0.35–0.38	0.02 ± 0.006	0.02–0.03
60	1.1 ± 0.04	1.04–1.16	1.59 ± 1.09	1.43–1.67	0.08 ± 0.009	0.07–0.09
80	0.58 ± 0.07	0.5–0.71	2.4 ± 0.16	2.19–2.61	0.81 ± 0.09	0.7–0.95
100	0.18 ± 0.04	0.11–0.24	4.94 ± 0.86	3.66–5.85	1.52 ± 0.59	1.1–2.55

Relative volumes are given as a percentage of the total puparial volume

**Table 8:** Classification matrix for the 25 test samples of *Lucilia sericata* based on the LDA model developed for *Calliphora vicina*

Percentage	10%	20%	30%	40%	50%	60%	70%	80%	90%	100%	Percentage correctly classified
20	0	**1**	*3*	*1*	0	0	0	0	0	0	20
40	0	0	*2*	**3**	0	0	0	0	0	0	60
60	0	0	0	0	0	**5**	0	0	0	0	100
80	0	0	0	0	0	0	0	**5**	0	0	100
100	0	0	0	0	0	0	0	0	*2*	**3**	60
Total	0	1	5	4	0	5	0	5	2	3	**68**

Column headings correspond to the estimated percentages and row headings to the actual percentages. Values showing correct classifications are presented in bold, and those showing misclassifications are presented in italics

## Discussion

### Qualitative analysis of tomographic data

Overall, the internal morphological features and the chronology of the changes observed in micro-CT virtual sections (Figs. 2, 3, 4 and 5; Table 1) are consistent with those described in previous studies of the blow fly intra-puparial period using traditional imaging techniques (e.g. [13, 19, 21, 23, 28, 31]). Indeed, age-informative morphological features observed with the current micro-CT scans had been corroborated with histological sections in a previous publication [16], confirming the great potential of micro-CT for visualising developmental processes [22]. Furthermore, the use of micro-CT allows the unambiguous determination of some informative developmental events, like the completion of the apolyses, which are crucial for a correct analysis based on internal morphological characters [14, 16]. With the current resolution, age-diagnostic qualitative characters can be determined for most 10% time intervals of the intra-puparial period which, moreover, can be used over a range of temperatures (Table 1) and even potentially applied to other blow fly species like *L. sericata* (Fig. 5). It must be noted that no possible discrimination between the 90 and the 100% stages of the total intra-puparial period is possible, neither on the basis of qualitative characters (Table 1) nor on quantitative analyses. In addition, special caution must be taken within the 50–60% stages of the total intra-puparial period. The change of shape of the lamina in horizontal section, from a horseshoe to an unfolded but not fully extended structure, is the most age-informative character at those intervals (Table 1). However, the observed variability within the 60% stage of the total intra-puparial period, where most specimens showed an unfolded but not fully extended lamina (Fig. 3h) but some showed the horseshoe shape typical of the 40–50% intervals (Fig. 3f, g), demands conservativism when using the shape of the lamina in the estimation of insect age. Thus, we recommend that an estimation of 50–60% of the total intra-puparial period should be suggested when the following combination of characters occurs: (1) lamina is horseshoe-shaped in horizontal section, (2) crop is located in the posterior part of the thorax and (3) the pre-helicoidal region of the midgut is tube shaped (Table 1). These conservative age estimates based on qualitative characters should then be complemented with quantitative measures of age whenever possible. Furthermore, it must be taken into account that insect evidence collected at the crime scene usually consists of many specimens of approximately the same age. Therefore, it is likely that an age estimate would be based on several puparial samples rather than just one, potentially narrowing the estimation in situations of uncertainty.

Currently, most published approaches to age estimation during the blow fly intra-puparial period rely strictly on a dissection of the puparium for an external morphological analysis of the insect inside (e.g. [20, 36, 37]). However, without disregarding the usefulness of such analyses, we must emphasise again that an internal morphological analysis involving a sectioning of the sample is essential for a correct interpretation and delimitation of the intra-puparial developmental stages [14]. The lack of an internal analysis is very likely behind several historical misuses and misinterpretations which can result in flawed ageing method analyses and, as already mentioned, lead to significant errors in _min_PMI estimations (see [14] for a review). In forensic entomology casework, internal morphological analyses of the puparial samples should be encouraged whenever possible, ideally in combination with an external analysis [19]. Nevertheless, considering the aforementioned drawbacks of traditional histological methods in the specific case of blow fly puparia [16, 21, 27], it can be expected that many forensic entomology practitioners may avoid its routine use. For example, Davies et al. [21] already suggested the usefulness of the morphological changes shown by the optic lobes during the intra-puparial period as qualitative markers of age. However, the histological sections of the brain presented in that paper are very fragmented and/or wrinkled and show a loss of significant parts of tissue, as clearly acknowledged by the authors [21]. This may result in misinterpretations or oversights of important structures like the lamina. The lamina is the last optic lobe to develop [33], and its appearance and shape are age informative (Table 1), so it is crucial to ensure a good quality of the sections in an internal morphological analysis. Our study confirms that micro-CT is a feasible, reliable and non-destructive alternative to traditional histology for qualitative internal morphological analyses of preserved puparial samples for _min_PMI estimations, as the preliminary results of Richards et al. [8] suggested.

Nevertheless, it must be emphasised that micro-CT does not achieve the level of spatial resolution of other imaging techniques like traditional histology or transmission electron microscopy [16]. With the current available resolution, micro-CT virtual sections cannot document changes at the cellular level [8, 16]. Moreover, iodine staining can sometimes result in blurring of the edges of some structures like the central brain neuropils [38] or in very bright tissues—note the occurrence of some bright artefacts within the fat bodies and undifferentiated tissues in Figs. 4a and 5c. Iodine certainly can overstain some mineralised tissues [22], for example the meconium and the fat bodies, which are rich in uric acid during metamorphosis [35]. For these reasons, the current study only determined unequivocal and easily identifiable morphological features for their use as qualitative markers of age (Table 1). Further research on optimal staining times and methods [39], together with continuous advances in micro-CT technology, might enable the use of additional age-informative characters in complex systems like the central brain or the reproductive organs. In this context, it is important to emphasise the importance of a proper training in insect morphology and development for forensic entomologists, in order to avoid misinterpretations of concepts and/or anatomical parts.

Finally, the issue with the explosion of the gas bubble in most puparia collected at the 10% stage of the total intra-puparial period must be briefly discussed. This explosion was very likely due to the high pressure generated during submersion in hot water (indeed, an explosion causing even the breaking of the puparium was observed during hot water fixation in other specimens collected at the same interval, but these specimens were not used for scanning). Earlier studies at 6 and 12 h old, when the gas bubble was smaller [16], did not result in an explosion—it was only seen from 18 to 24 h old, when the gas bubble was larger. Hot water fixation prior to storage in 80% ethanol is recommended for the blow fly immature stages collected at the forensic scene as it avoids the marked decomposition of tissues caused by direct placement in ethanol and thus enables a proper analysis of the sample [10, 27, 40]. In fact, Richards et al. [8] already identified decomposition as a potential problem when using live puparial specimens placed directly in ethanol. However, we should not overestimate the problem because the gas bubble is only present during the first 10% of the total intra-puparial period and, only for a short time then, is sufficiently large to ‘explode’ [16]—all of our 10% samples featured a large bubble. Therefore, its potential explosion in a given sample may not be critical for estimating the age because, either exploded or not, it should be easy to assign the specimen to that development interval on the basis of a qualitative analysis (Fig. 2). Nevertheless, the explosion of the gas bubble can preclude further morphological analyses of the sample in developmental studies and therefore alternative fixation methods should be tested.

### Quantitative analysis of tomographic data

The current study highlights the suitability of using quantitative measures of organ development for improving age estimates during the blow fly intra-puparial period. This is supported by the lack of significant differences between the three experimental rearing temperatures in the changes in volume of the three analysed structures (indirect flight muscles, pre-helicoidal region of the midgut and rectal pouch) during the intra-puparial development. Nevertheless, as highlighted in the “Results” section, there were significant differences in the volume of the indirect flight muscles between the 15 and 24 °C experimental set-ups at the 70% stage of the total intra-puparial period (Fig. 6a). Although a real effect of the temperature on the relative developmental rate of that organ system should not be discarded, it must be taken into account that the number of measured samples (15 per developmental interval, 5 per each experimental temperature) is relatively low. Therefore, the observed differences at 70% of the total intra-puparial period might be simply an artefact due to the low sample size and, to this, being a period of dramatic change over a short period of time (Fig. 6c), especially considering that the ranges of the volume values overlapped among temperatures (Fig. 6a). Further research using more experimental temperatures, replicates and samples might throw more light on this issue.

On the other hand, the accuracy of the classifiers created using a LDA for *C. vicina* was overall high for both the samples used to fit the model (Table 5) and the additional test samples (Table 6). Nevertheless, the occurrence of some misclassifications based only on quantitative analyses of tomographic data (particularly between the 20–30 and the 90–100% stages of the total intra-puparial development; see Table 5) also highlights the demand for conservative age estimates because of overlap with adjacent development intervals. Similar conservative age estimates are also demanded by alternative ageing methods like external morphological and gene expression analyses [20, 24]. In many situations with our tomographic data, such as discriminating between the 20 and 30% stages of the total intra-puparial period, ambiguity could be solved by combining both quantitative and qualitative analyses (Table 1). However, in discriminating between the 90 and 100% stages of the total intra-puparial period, there are major problems because distinction based on a qualitative analysis is not possible either (Table 1). It would appear that a pharate adult at the 90% stage of intra-puparial development is, from a micro-CT perspective, fully formed. Nevertheless, some changes not visible to micro-CT could still be occurring. For example, during the development interval from 90 to 100% stages, the pharate adult completes the tanning and darkening of some external structures [20]. Furthermore, the 100% stage corresponds to the time when most adults are expected to emerge from the puparium but, due to natural variation, it is certainly possible that some individuals could emerge earlier; indeed, some adults from the experimental batches did emerge between 90 and 100%. As mentioned above, further research on optimal staining methods, together with further advances in micro-CT technology, may provide sufficient resolution to incorporate additional organ system measurements to the LDA model, thus improving the accuracy of the classifiers.

LDA had been previously used in forensic entomology to determine the larval instar of forensically relevant beetles of families Silphidae and Staphylinidae with great success [41, 42]. In these cases, percentages of correct classifications close or equal to 100% were achieved using larval instar classifiers at the subfamily, genus or species levels [41, 42]. As larval growth in these Coleoptera groups is not a continuous process, they are ideal subjects for LDA classifications. Silphidae and Staphylinidae larvae are highly sclerotised, and the different instars are separated by the ecdysis or moulting of the old cuticle, followed by a short growth phase before the new cuticle hardens and darkens. In contrast, the development of the indirect flight muscles and the alimentary canal during the whole blow fly intra-puparial period is essentially a continuous process, even if some marked bursts of volume increase can be observed—particularly in the indirect flight muscles and the rectal pouch during the last intervals of the intra-puparial period—the overlap of volumes between consecutive intervals is the general pattern (Fig. 6b–d). Indeed, the classification of beetle larval instars during the post-ecdysis short growth phases can be more problematic and lead to some misclassifications as belonging to the previous larval instar [41].

The current results suggest that a classifier developed for one species might not be valid for another species. This was, at least, the case when the classifier developed for *C. vicina* was used for another calliphorid species, *L. sericata* (Table 8). Developing larval instar classifiers for several Silphinae species belonging to different genera, Frątczak and Matuszewski [42] showed that classifiers at the subfamily or genus level could achieve a high accuracy for some species. Future studies using other forensically relevant species belonging to the same subfamily and/or genus as *C. vicina*, e.g. *Calliphora vomitoria* (Linnaeus), might show if classifiers developed for one species can be applied successfully to closely related species to age the blow fly intra-puparial period.

## Conclusions

Our study confirms and highlights micro-CT as a promising method for providing accurate and reliable age estimations of blow fly samples during their intra-puparial period [8]. In practical terms, ageing blow flies to within 10% of the intra-puparial period is probably as much as could be realistically expected in a criminal investigation, bearing in mind the natural variation in developmental rates within any generational cohort of larvae and the conservative confidence intervals that would need to be placed on any estimation of age due to difficulties in estimating the many variables at a scene, for example the temperatures to which the insects were exposed during their development [43]. Moreover, as mentioned above, the development of most organs during metamorphosis is essentially a continuous process, not stepped at 10% intervals. Therefore, it must be expected that some individuals in a forensic sample will show morphological characters that are intermediate between those described here and so could not be assigned to a single interval.

Richards et al. [8] suggested using a combination of both internal and external morphological markers to achieve a higher degree of precision of age estimates. Iodine is not a permanent stain, so after being scanned in micro-CT, specimens may be suitable for a subsequent analysis of external morphological characters [8, 19, 20] if they are first properly washed. Further research should test whether the puparial samples fixed and prepared for micro-CT scanning might also be suitable for later molecular analyses [24, 27]. Certainly, the application of both quantitative and qualitative micro-CT internal morphological analyses in combination with external morphological [8, 19, 20] and gene expression [24] analyses would improve the degree of accuracy and precision, by not only potentially narrowing but also corroborating the age estimates provided by different methods. An ideal future scenario for analyses of blow fly puparia collected at the forensic scene would involve a preliminary, non-invasive internal analysis using micro-CT. Scanning using the same settings as in the current study is a relatively rapid process (∼40 min), with the posterior automatic reconstruction, manual rendering and first visual analysis taking 2–3 h. This is in contrast to traditional histology, where the sectioning (obviously limited to one single plane), mounting and staining of a whole single specimen can take 1 day at best and many times with unsatisfactory results (see the “Discussion” above). With micro-CT, however, several specimens can be scanned and reconstructed at the same time, saving time and money [8], and the same specimen can be virtually dissected in any plane [8, 16]. Then, if needed, a further quantitative analysis of the tomographic data (which can take several hours) and/or invasive external morphological and gene expression analyses could be conducted. Scanning the samples before performing more invasive analyses has an additional advantage: the tomographic data can be retrieved and reanalysed at any time in the future if a particular case needs to be reassessed [3] and can be made available to both the defence and the prosecution for independent analyses [8]. Furthermore, there is no reason why puparial samples from forensic exhibits could not be stained and scanned even years after collection if they had been properly fixed and preserved [27].

Finally, we cannot ignore the reality that a major current drawback in the use of micro-CT is its limited availability to forensic researchers and practitioners, as it is still regarded as an emerging technology [6]. Nonetheless, given its wide range of potential roles in the forensic practice beyond entomology [6], it is very likely that micro-CT will continue to be progressively implemented across forensic and research institutions around the world (and the associated costs will consequently decrease), in a similar manner to that whereby CT became a routine method in post-mortem investigations [3].
